# Paternal and maternal use of dupilumab in patients with atopic dermatitis: a case series

**DOI:** 10.1111/ced.14725

**Published:** 2021-06-04

**Authors:** A. L. Bosma, L. A. A. Gerbens, M. A. Middelkamp‐Hup, P. I. Spuls

**Affiliations:** ^1^ Department of Dermatology, Amsterdam University Medical Centers (Amsterdam UMC), location Academic Medical Center Amsterdam Public Health, Immunity and Infections University of Amsterdam Amsterdam The Netherlands

## Abstract

Dupilumab is a relatively new treatment option for patients with moderate to severe atopic dermatitis. There is a lack of knowledge about the effects of treatment with dupilumab during conception for both men and women, as well as during pregnancy and lactation in women. We report four patients (two men, two women) who expressed a wish to conceive during treatment with dupilumab in daily practice. Both men conceived during dupilumab treatment, while the two women discontinued dupilumab because of anticipated pregnancy. Apart from disease flares in both of the patients who discontinued treatment, no complications were reported concerning the ability to conceive, the course of the pregnancy or the fetal outcome. We present an overview of the current available literature on dupilumab during conception, pregnancy and lactation, which can guide considerations for patients on dupilumab wishing to conceive a child. Until more data are available, preference should be given to treatment with topical corticosteroids, phototherapy, systemic corticosteroids and ciclosporin.

Atopic dermatitis (AD) is a chronic, pruritic inflammatory skin disorder. Moderate to severe cases, requiring systemic immunomodulating therapy, include patients of reproductive age. A lack of knowledge exists on the effects of dupilumab treatment during conception in both men and women, as well as during pregnancy and lactation in women. In this case series, we present two men who conceived during treatment and two women who discontinued dupilumab due to anticipated pregnancy.

## Report

Patients were all diagnosed with AD based on the UK Working Party criteria and gave consent for participation in the TREAT NL (TREatment of ATopic eczema, the Netherlands) registry, before starting on‐label dupilumab in the context of routine clinical practice at the Amsterdam University Medical Centers.^1^ All patients had moderate to severe disease (mean Eczema Area and Severity Index 21.6) and were allowed to use concomitant systemic or topical treatments. Further patient characteristics are summarized in Table 1.

**Table 1:** Patient characteristics.

Patient	Sex	Age, years		Atopic comorbidities^a^	Obstetric history	Severity scores at baseline^b^ (and restart dupilumab)	Concomitant systemic therapy at baseline^b^ and during follow‐up
		Patient	Partner				
1	F	29	30	Asthma, allergic rhinoconjunctivitis, food allergies, allergic contact dermatitis	Uncomplicated	EASI: 7.1 (21.3); POEM: 24 (26); DLQI: 11 (18)	Baseline: none Follow‐up: NB‐UVB phototherapy for 18 weeks before pregnancy and during dupilumab discontinuation
2	F	31	29	Asthma, allergic rhinoconjunctivitis, food allergies, allergic contact dermatitis	Uncomplicated	EASI: 32.2 (–); POEM: 27 (27); DLQI: 27 (11)	Baseline: prednisolone 30 mg/day in a tapering schedule for 39 days Follow‐up: ciclosporin 250–300 mg/day for 20 weeks and prednisone 5–30 mg/day for 8 weeks during pregnancy and during dupilumab discontinuation
3	M	34	32	Allergic rhinoconjunctivitis	Uncomplicated	EASI: 34.6; POEM: 21; DLQI: 7	Baseline: none Follow‐up: none
4	M	26	27	None	Uncomplicated	EASI: 12.4; POEM: 28; DLQI: 27	Baseline: prednisolone 5 mg/day for 1 day Follow‐up: none

DLQI, Dermatology Life Quality Index (0–30); EASI, Eczema Area and Severity Index (0–72); NB‐UVB, narrowband ultraviolet B; POEM, Patient‐Oriented Eczema Measure (0–28).

^a^ Physician‐assessed diagnosis of the following comorbidities: asthma, allergic rhinoconjunctivitis, atopic eye disease, eosinophilic oesophagitis, food allergies and allergic contact dermatitis;

^b^ defined as start of dupilumab treatment.

Data collection was performed using a predefined case record form, including paternal and maternal use of medication (defined as the use of systemic therapy in anticipation of conception). After careful consideration by physicians and patients, one man received dupilumab while attempting to conceive, while two women discontinued in anticipation of pregnancy. A second man conceived unexpectedly. Both of the women were advised to use contraception for at least 3 months prior to conception, as the median time for dupilumab concentrations to fall below the lower limit of detection after discontinuation is 10–11 weeks.^2^

At 12 weeks after starting dupilumab, the first man expressed an active wish to father a child. Conception was successful at 33 weeks of treatment, resulting in the couple’s first pregnancy. The second man unexpectedly conceived at 69 weeks of treatment. Both patients continued on dupilumab, resulting in sufficient disease control during conception. Both of the partners were healthy. Both pregnancies proceeded without complication and the neonates, both born at or close to term, were healthy.

The first woman discontinued dupilumab after 36 weeks of treatment, and conceived 34 weeks later. During the interval between treatment discontinuation and pregnancy, she received narrowband ultraviolet B phototherapy for 18 weeks. During pregnancy, her AD flared and was controlled with topical corticosteroids. After a full‐term uncomplicated pregnancy, the patient delivered a healthy baby. She decided to breastfeed and her AD was well‐controlled for a week, flaring thereafter. After stopping breastfeeding, the patient restarted dupilumab with a loading dose, 7 months after delivery.

The second woman conceived a child 16 weeks after discontinuation of a 25‐week dupilumab course. She experienced a severe flare at 30 weeks after discontinuation (14 weeks’ gestation) for which she was started on treatment with bleach baths, emollients and potent topical steroids at our daycare unit. A week later (15 weeks’ gestation) she was started on ciclosporin 250 mg/day (3.4 mg/kg/day). Fourteen weeks thereafter, concomitant treatment with prednisolone 30 mg/day was deemed necessary. After consultation with her obstetrician, the patient was induced at 37 weeks, due to the physical and mental distress resulting from AD. The patient delivered a healthy baby, without complication. She refrained from breastfeeding in order to restart dupilumab. A loading dose of dupilumab was administered 2 days after delivery, resulting in complete relief of her symptoms within 3 weeks.

No published human clinical studies are available on dupilumab during pregnancy, breastfeeding or conception.^3^ Pregnant or breastfeeding women, or those planning to conceive, were excluded from dupilumab trials.^2^ Animal studies have shown no fetal abnormalities or impacts on fertility.^1^ The European Medicines Agency reports that the spontaneous abortion rate in patients treated with dupilumab did not exceed that in the general population.^2^

In accordance with other atopic diseases, AD can worsen during pregnancy and impart a risk of serious infection.^3^, ^4^ Atopic diseases have shown to be associated with reduced fertility.^3^ Both pregnancy and atopic diseases are characterized by T helper (Th)2 upregulation.^3^, ^5^ Interestingly, considering the shift towards Th2 cell differentiation, targeting interleukin‐4 with dupilumab has been suggested as potential treatment option for pregnant patients with Th2‐dominant diseases, such as AD, to induce the Th1/Th2 balance and improve disease.^5^ However, as this theory has never been investigated in pregnancy, it warrants further research, in particular on the risk of spontaneous abortion.^5^

Two previous case reports have been published on dupilumab in pregnancy and lactation.^6^, ^7^ The first case concerned a patient who discontinued dupilumab at 2 weeks of gestation, followed by reintroduction at 20 weeks.^6^ In the second case dupilumab was initiated at 24 weeks of gestation, in addition to treatment with prednisone.^7^ Both patients delivered a healthy baby, without complications, at 40 and 37 weeks of pregnancy, respectively. The first patient breastfed her infant while receiving dupilumab, during an uncomplicated observation period of 4 months.

It is unknown whether dupilumab is excreted in breast milk or whether systemic absorption occurs after ingestion. Because dupilumab is a large protein molecule, the amount in breast milk is expected to be low, and absorption is unlikely because it is probably destroyed in the infant's gastrointestinal tract.^8^ However, the molecule size does not prevent placental transfer.^3^, ^4^, ^7^

The Summary of Product Characteristics (SmPC) for dupilumab indicates that it is preferable to avoid the drug in pregnancy, unless a physician advises it and only if the potential benefit justifies the potential risks.^1^ In addition, a decision must be made whether to discontinue breastfeeding or dupilumab, taking into account the benefits for mother and child.^1^ The SmPC does not mention any restrictions for men taking dupilumab who wish to father a child.^1^

The European Task force on Atopic Dermatitis advises avoiding dupilumab by pregnant or breastfeeding women until there is more experience. They report that there is no literature on men taking dupilumab who wish to father a child, and that theoretically there could be a transfer of dupilumab to the seminal fluid.^9^ For both sexes, preference is given to treatment with topical corticosteroids, phototherapy, systemic corticosteroids, ciclosporin and, only in select cases, azathioprine, if a child is desired.^4^, ^9^ Owing to the lack of evidence, guidelines recommend avoiding dupilumab during pregnancy and lactation.^10^ Absolute contraindications exists for treatments such as methotrexate and mycophenolate mofetil.^4^, ^9^ Similarly to dupilumab, there are few human data available for emergent treatments such as Janus kinase inhibitors and other biologics, resulting in negative recommendations for use during conception and pregnancy.^4^

In practice, patients are usually reluctant to discontinue treatment with dupilumab. These concerns are justified, as we observed disease flares in the two patients who discontinued and disease control in the two patients who continued. It is important to take into consideration that absence of adequate treatment can also involve adverse effects for mother, father and child.

In conclusion, we observed uncomplicated pregnancies in two women with AD who discontinued dupilumab prior to conception, and two female partner pregnancies of men with AD treated with dupilumab during conception. More data are required from large prospective cohort studies to detect adverse events. At the moment, two registries are planned for dupilumab in pregnancy (R668‐AD‐1639, R668‐AD‐1760).^2^ In addition, we will continue data collection in the TREAT NL registry.
